# Vesicular Trafficking Systems Impact TORC1-Controlled Transcriptional Programs in *Saccharomyces cerevisiae*

**DOI:** 10.1534/g3.115.023911

**Published:** 2016-01-06

**Authors:** Joanne M. Kingsbury, Maria E. Cardenas

**Affiliations:** Department of Molecular Genetics and Microbiology, Duke University Medical Center, Durham, North Carolina 27710

**Keywords:** Golgi-to-endosome trafficking, rapamycin mechanism of action, sulfur-containing amino acid metabolism genes, ribosome biogenesis genes, phosphate-responsive network

## Abstract

The Target of Rapamycin Complex I (TORC1) orchestrates global reprogramming of transcriptional programs in response to myriad environmental conditions, yet, despite the commonality of the TORC1 complex components, different TORC1-inhibitory conditions do not elicit a uniform transcriptional response. In *Saccharomyces cerevisiae*, TORC1 regulates the expression of nitrogen catabolite repressed (NCR) genes by controlling the nuclear translocation of the NCR transactivator Gln3. Moreover, Golgi-to-endosome trafficking was shown to be required for nuclear translocation of Gln3 upon a shift from rich medium to the poor nitrogen source proline, but not upon rapamycin treatment. Here, we employed microarray profiling to survey the full impact of the vesicular trafficking system on yeast TORC1-orchestrated transcriptional programs. In addition to the NCR genes, we found that ribosomal protein, ribosome biogenesis, phosphate-responsive, and sulfur-containing amino acid metabolism genes are perturbed by disruption of Golgi-to-endosome trafficking following a nutritional shift from rich to poor nitrogen source medium, but not upon rapamycin treatment. Similar to Gln3, defects in Golgi-to-endosome trafficking significantly delayed cytoplasmic–nuclear translocation of Sfp1, but did not detectably affect the cytoplasmic–nuclear or nuclear–cytoplasmic translocation of Met4, which are the transactivators of these genes. Thus, Golgi-to-endosome trafficking defects perturb TORC1 transcriptional programs via multiple mechanisms. Our findings further delineate the downstream transcriptional responses of TORC1 inhibition by rapamycin compared with a nitrogen quality downshift. Given the conservation of both TORC1 and endomembrane networks throughout eukaryotes, our findings may also have implications for TORC1-mediated responses to nutritional cues in mammals and other eukaryotes.

The conserved Target of Rapamycin Complex I (TORC1) pathway is a central processing conduit that senses and responds to myriad environmental signals, arresting growth under nutrient starvation and stress conditions, and reestablishing growth when favorable conditions are restored. Activation of TORC1 in optimal growth conditions drives protein and ribosome biosynthesis via the Sch9 kinase, and the transcription factors Sfp1, Fhl1, Stb3, Maf1, and Dot6/Tod6 (Upadhya *et al.* 2002; Jorgensen *et al.* 2004; Marion *et al.* 2004; Schawalder *et al.* 2004; Urban *et al.* 2007; Liko *et al.* 2007; Huber *et al.* 2009, 2011; Lempiainen *et al.* 2009; Lippman and Broach 2009). Concomitantly, activated TORC1 directly inhibits Atg1/13 to block autophagy (Kamada *et al.* 2010), and stimulates the formation of the Tap42-Sit4 complex, thereby preventing the expression of the nitrogen catabolite-repression (NCR), stress-responsive, and retrograde pathway genes regulated by the transactivators Gln3, Gat1, Msn2/4, and Rtg1/3 (Di Como and Arndt 1996; Beck and Hall 1999; Crespo *et al.* 2002; Duvel *et al.* 2003; Santhanam *et al.* 2004). Conversely, TORC1 activity is inhibited upon: 1) starvation for amino acids, nitrogen, glucose, or phosphate; 2) osmotic, heat, or oxidative stress; or 3) rapamycin treatment (Cardenas *et al.* 1999; Hardwick *et al.* 1999; Beck and Hall 1999; Urban *et al.* 2007; Loewith and Hall 2011; Takahara and Maeda 2012; Yan *et al.* 2012; Panchaud *et al.* 2013). TORC1 inactivation by rapamycin or nitrogen starvation results in dissociation of the TORC1-Tap42-Sit4 interactions, releasing Tap42-Sit4 into the cytosol, which, in turn, results in the dephosphorylation and dissociation of the Gln3-Ure2 complex, thereby enabling Gln3 relocation from the cytoplasm to the nucleus (Beck and Hall 1999; Yan *et al.* 2006). However, in poor nitrogen sources, such as proline, Sit4-dependent Gln3 translocation does not consistently reflect the level of Gln3 dephosphorylation (Cox *et al.* 2004; Tate *et al.* 2006), and apparently occurs despite a lack of dissociation of Tap42-Sit4 from membranes (Di Como and Jiang 2006). Furthermore, despite the common role of TORC1 in controlling these responses, each TORC1-inhibitory condition, including glucose starvation, osmotic-, oxidative- and heat-stress, results in distinct patterns and degrees of inhibition or activation of the different TORC1-regulated signaling branches, thus leading to distinct transcriptional profiles (Hughes Hallett *et al.* 2014).

Recent research underscores the many and varied roles that the vesicular membrane network contributes to enable TORC1 signaling. First, the vacuolar (yeast) or lysosomal (mammals) membrane acts as a scaffold, facilitating interactions between the TORC1 complex, its upstream modulators, and downstream effectors, many of which reside on this membrane (Cardenas and Heitman 1995; Zoncu *et al.* 2011; Huh *et al.* 2003; Wedaman *et al.* 2003; Araki *et al.* 2005; Gao and Kaiser 2006; Buerger *et al.* 2006; Urban *et al.* 2007; Sturgill *et al.* 2008; Wang *et al.* 2015). Indeed, translocation to the lysosomal membrane is required for mammalian (m)TORC1 activation (Sancak *et al.* 2008, 2010), while, under certain conditions, sequestration from the vacuole into stress granules inhibits TORC1 activity in both yeast and mammals (Takahara and Maeda 2012; Wippich *et al.* 2013; Thedieck *et al.* 2013). Second, a screen to identify new components of the TORC1 pathway revealed multiple genes involved in vesicular trafficking including: 1) all of the members of vacuolar protein sorting class C (Vps-C) complexes, which mediate vacuole–vacuole, vacuole–endosome docking, and fusion; and 2) EGOC GTPase components that mediate TORC1 activation in response to amino acids (Zurita-Martinez *et al.* 2007). Vacuoles are important amino acid reservoirs, and Vps-C mutants exhibit marked defects in amino acid homeostasis (Banta *et al.* 1988; Kitamoto *et al.* 1988; Raymond *et al.* 1992). TORC1 signaling in Vps-C mutants is severely compromised, due in part to defects in amino acid homeostasis and reduced EGOC–TORC1 interaction (Zurita-Martinez *et al.* 2007; Kingsbury *et al.* 2014). Similarly, perturbation of mammalian Rab GTPase Rab5 and hVps39 levels affects mTORC1 activity in response to amino acids and insulin, and this effect was attributed to reduced mTORC1-Rheb interactions (Flinn and Backer 2010; Flinn *et al.* 2010).

In a further association between the vesicular trafficking system and TORC1 signaling, Vps-C and Vps-D mutations, which disrupt Golgi-to-endosome trafficking, were found to perturb TORC1-regulated NCR gene expression and Gln3-nuclear translocation when cells were shifted from rich medium to proline as the nitrogen source, but not when cells were exposed to rapamycin (Puria *et al.* 2008). In essence, similar results were reported elsewhere, showing a Golgi-to-endosome trafficking requirement for Gln3 nuclear translocation upon a nitrogen source downshift, but also upon rapamycin treatment under certain growth conditions (Fayyadkazan *et al.* 2014). These results are consistent with a model whereby under TORC1-inhibitory conditions, such as growth in proline medium where Tap42-Sit4 is associated with TORC1 at the vacuole (Di Como and Jiang 2006), association of the Gln3-Ure2 complex with Golgi-derived vesicles is important for mediating its dephosphorylation by Tap42-Sit4 to enable Gln3 dissociation and nuclear translocation (Puria *et al.* 2008; Puria and Cardenas 2008).

TORC1-controlled transcriptional programs were largely characterized as those perturbed by exposure of cells to rapamycin under rich growth conditions. Thus, it is important to compare the rapamycin-dependent transcriptional programs with those operating under conditions where TORC1 is inhibited by a nutritional down-shift. In this study, we sought to characterize the full extent by which defects in the vesicular trafficking system impact TORC1-regulated transcriptional programs. Using microarray analyses, we compared expression profiles between the WT and a Vps-D mutant (*vps45*) grown in rich medium and exposed or not to rapamycin, or upon a shift to the poor nitrogen source proline. Consistent with our earlier findings (Puria *et al.* 2008), a greater proportion of genes were differentially regulated between the *vps45* mutant and the WT when these strains were grown in the presence of proline *vs.* rapamycin exposure in rich medium. Functional categories particularly enriched among those genes differentially regulated between the WT and *vps45* mutant in proline included the NCR genes, as previously identified (Puria *et al.* 2008), and genes involved in the metabolism of phosphate and methionine/sulfur amino acids, ribosomal protein (RP) production and ribosome biogenesis (Ribi), as well as the mating and pheromone response genes, which are regulated independently of TORC1. Our results therefore reveal differences in the TORC1-regulated programs elicited by rapamycin exposure under growth in rich medium compared with growth in poor nitrogen sources, and underscore the contributions of Golgi-to-endosome trafficking to these responses.

## Materials and Methods

### Strains, media, and growth conditions

Standard yeast growth media consisted of Yeast Extract Peptone Dextrose (YPD), Synthetic Dextrose (SD), Synthetic Complete (SC), or variations of either synthetic medium with various amino acids and supplements omitted or added to complement auxotrophies or select for plasmid maintenance (Sherman *et al.* 1974). Synthetic (S) proline medium consisted of SD supplemented with 1 g/L proline instead of ammonium sulfate. When required, media was supplemented with 100 nM rapamycin (LC Laboratories).

All strains used in this study were isogenic with BY4741 or BY4742 (Brachmann *et al.* 1998), and are listed in Supporting Information, Table S1. Typically, strains containing genes disrupted with the kanMX4 marker were obtained from the *Saccharomyces* Genome Deletion Project (distributed by Invitrogen, Carlsbad, CA) (Giaever *et al.* 2002), and mutations were confirmed by PCR with appropriate DNA primers. All yeast transformations were performed using the lithium-acetate-mediated method (Gietz *et al.* 1995). All gene disruptions were confirmed by PCR. Plasmids and primers employed in this study are listed in Table S2 and Table S3, respectively.

### TORC1 kinase assay

For analysis of Sch9 phosphorylation, protein was extracted following trichloroacetic acid treatment of cell cultures, and western blot analysis employing Sch9 and phospho-Thr737-Sch9 antibodies was performed as described previously (Kingsbury *et al.* 2014).

### Microarray analysis

For microarray expression analysis, the WT and *vps45* mutant were grown overnight in YPD medium, and diluted in 100 ml fresh YPD to an OD_600nm_ of 0.15. Cells were incubated until the OD_600nn_ reached 0.8, and cultures were divided for three treatments. Drug vehicle (1 µl/ml of 95% ethanol, 5% Tween 20) and 100 nM rapamycin was added for the first and second 30-ml treatments, respectively, and cells were incubated for a further 30 min. For the third treatment, cells were washed twice with S-proline+leu+lys+ura+his medium, resuspended in 30 ml of the same medium, and incubated for a further hour. At the end of the incubations, cells were immediately harvested, and cell pellets were snap-frozen in a dry ice-ethanol bath and stored at –80° until RNA isolation. RNA was extracted using the RNeasy Mini Kit (Qiagen), and treated with DNaseI (Ambion) according to manufacturer’s instructions. Transcript expression profiles were generated by hybridization to GeneChip Yeast Genome 2.0 arrays (Affymetrix, Santa Clara, CA). All processing of RNA samples for microarray analysis (cDNA synthesis, labeling, hybridization, and data management) was performed by the Duke Microarray Facility according to their standard procedures (http://www.genome.duke.edu/cores/microarray/). The dataset was normalized and analyzed using Partek Genomics Suite software. For hierarchical clustering, transcripts that differed at least twofold between the different conditions were normalized to a mean of 0 and SD of 1, and analyzed using Pearson’s dissimilarity mode of normalization. All ANOVA data are included in Table S8. Experiments were performed in triplicate.

### Quantitative real time (RT)-PCR analysis

Analysis of individual transcripts was performed using RT-PCR. cDNA was synthesized from 1 µg RNA, extracted as described above, using the AffinityScript QPCR cDNA Synthesis Kit (Agilent Technologies), followed by RT-PCR using the Brilliant III Ultra Fast SYBR Green QRT-PCR Master Mix (Agilent Technologies), according to the manufacturer’s instructions. RT-PCR reactions were analyzed using a StepOne Real-Time PCR System with StepOne 2.1 software (Life Technologies). *ACT1* was used as an internal control for all experiments.

### Microscopy

The subcellular localization of GFP-tagged transcription factors was determined from strains grown as described for the microarray analysis. Following incubation, cells were concentrated by centrifugation, and applied to slide mounts containing 2% (w/v) agarose in the appropriate growth media. Cells were visualized and photographed using a Zeiss Axioskop 2 Plus microscope and AxioVision 4.6 image acquisition software. Exposure settings included 133% for 5 s or 100–200 ms for Met4-GFP or Nab2-NLS-2mCherry, respectively. To determine subcellular localization, we manually scored for nuclear concentration of the GFP signal colocalizing with the nuclear marker Nab2-NLS-mCherry for at least 100 cells from three or more fields of view for which no enhancement of image contrast was performed. Results presented are the average of at least two independent experiments.

### Data and reagent availability

All strains (Table S1) and plasmids (Table S2) constructed in this study are available on request. Table S3 lists all oligonucleotides used in this study. Expression data for methionine/sulfur amino acid metabolism, RP, Ribi, and stress response genes are listed in Table S4, Table S5, Table S6, and Table S7, respectively. Table S8 contains the complete ANOVA comparison of all expression data generated in this study.

## Results and Discussion

### Identification of TORC1-controlled transcriptional programs affected by perturbation of Golgi-to-endosome trafficking

**Figure 1:**
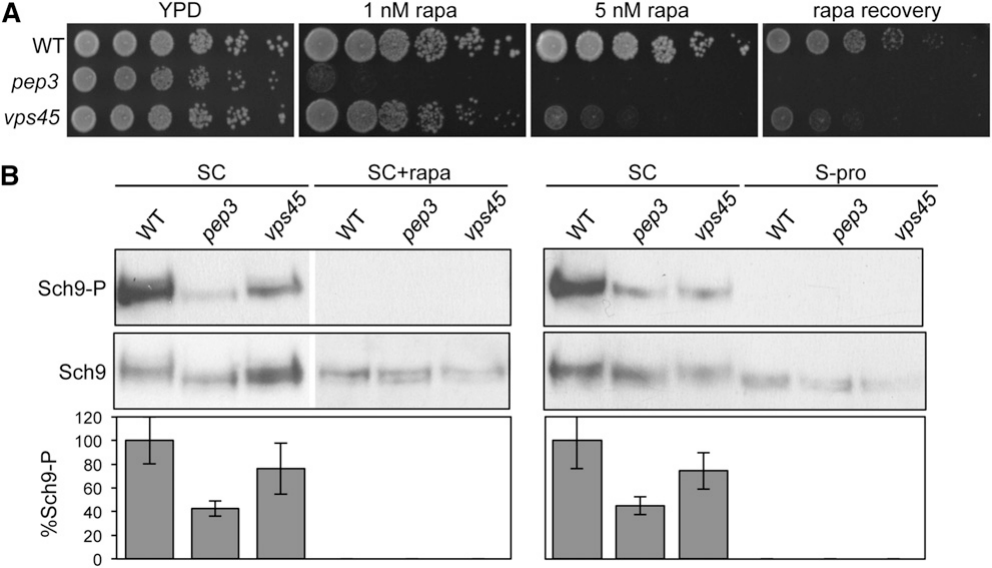
TORC1 signaling-relevant phenotypes are less severe for Vps-D compared with Vps-C mutants. (A) Rapamycin sensitivity and recovery of *pep3* and *vps45* mutants. Strains expressing plasmid-based HA_3_-*SCH9* with all auxotrophic mutations complemented were grown to exponential phase in SC-ura-his-lys-leu+gln. Cultures were divided in half and treated with either drug vehicle (control) or 200 nM rapamycin for 6 hr. The rapamycin-treated cells were washed two times with water, serially diluted fivefold, and 5 µl volumes of cells were applied onto YPD medium to test for recovery from rapamycin-mediated growth arrest. Drug vehicle control cultures were similarly diluted and plated on YPD without or with 1 or 5 nM rapamycin. Plates were photographed following 3- (YPD, 1 nM rapa) or 4-days (5 nM rapa) incubation. Results are representative of three independent experiments. (B) TORC1 kinase activity of *pep3* and *vps45* mutants. Strains described and grown as in (A) were harvested following no treatment (SC), 200 nM rapamycin addition for 30 min (SC+rapa), or in S-proline for 1 hr (S-pro). TORC1 activity was assessed by monitoring the phosphorylation of Sch9 Thr737, and overall Sch9 levels, by western blot with the anti-phospho-Thr737-Sch9 (Sch9-P), and anti-732-743-Sch9 (Sch9) antibodies, respectively. Phosphorylation levels were expressed as an average ratio of phosphorylated Sch9-P to Sch9 levels from three independent experiments, with error bars depicting the SD from the mean.

Vps-C complexes function in the docking and fusion of vesicles, endosomes, autophagosomes, and vacuoles at virtually all stages of the secretory pathway, while the Vps-D proteins are restricted to assisting vesicle trafficking between the Golgi and endosomes (Raymond *et al.* 1992; Srivastava *et al.* 2000; Peterson and Emr 2001). Both Vps-C and Vps-D mutants exhibit similar defects in Gln3 and Gat1 nuclear translocation, and NCR gene activation in the presence of proline as a nitrogen source (Puria *et al.* 2008; Fayyadkazan *et al.* 2014). We tested the effects of Vps-C and Vps-D mutations on TORC1 activity. Consistent with our previous findings (Puria *et al.* 2008), the *vps45* (Vps-D) mutants were more rapamycin-sensitive, had reduced recovery from rapamycin-induced growth arrest (Figure 1A), and displayed reduced phosphorylation of the TORC1 target Sch9, as compared with the wild-type (WT) (Figure 1B). In accord with a broader role of the Vps-C proteins in vesicle trafficking, the *vps45* TORC1-signaling-relevant phenotypes were considerably less severe than for the *pep3* (Vps-C) mutant.

Our previous studies identified defects in expression of TORC1-controlled NCR transcriptional programs when cells with defective vesicular trafficking were shifted from an optimal to a suboptimal nitrogen source (proline), but not following rapamycin exposure (Puria *et al.* 2008). We therefore sought to determine the full impact of the vesicular trafficking system on TORC1-controlled transcriptional programs.

**Figure 2:**
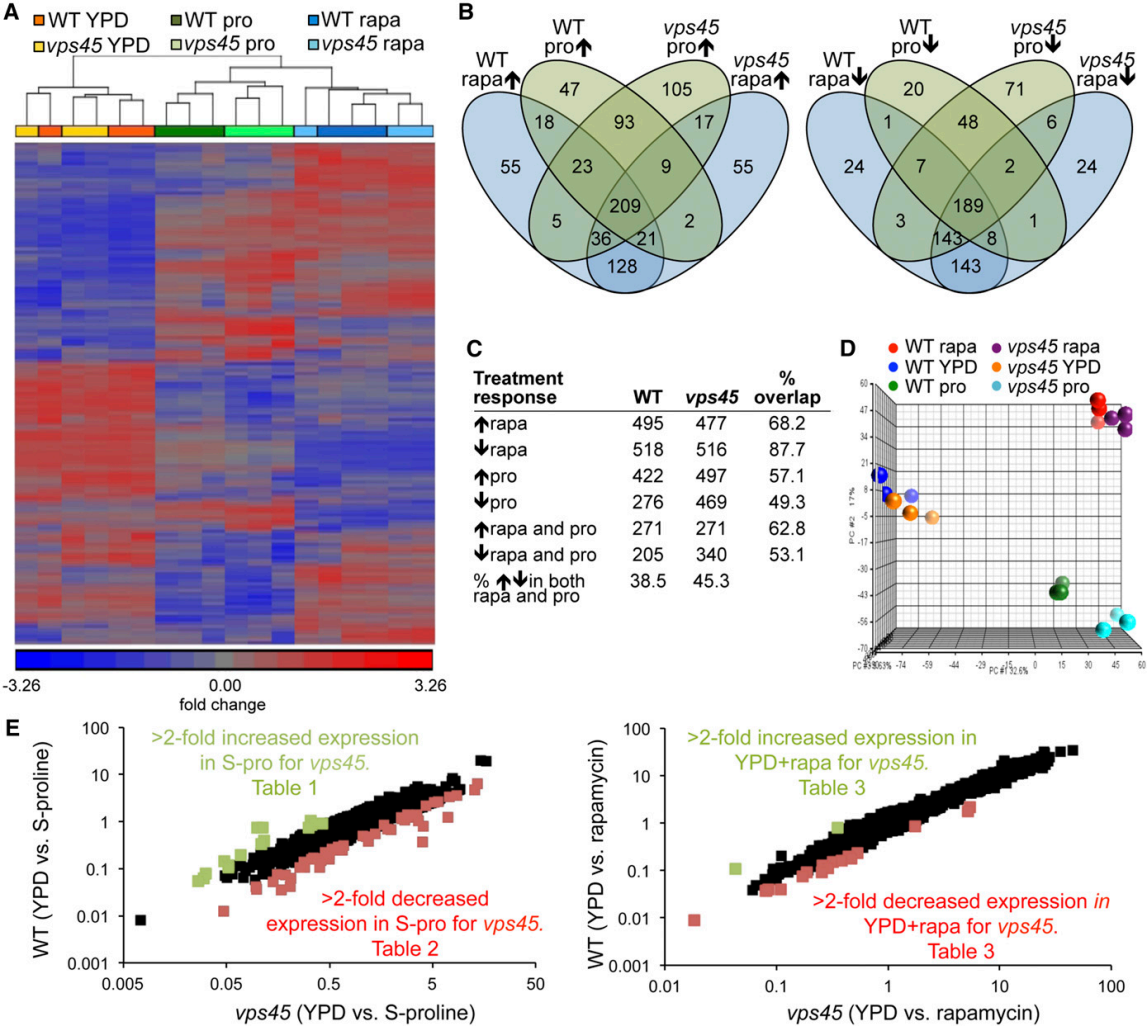
Vps-D mutation alters the transcriptional response to a switch to the poor nitrogen source proline. (A) Hierarchical analysis of transcript levels at least twofold differentially expressed in rich medium (YPD), 30 min rapamycin treatment (+rapa), and 1 hr following a shift from YPD into proline medium. Gene expression was standardized to a mean of 0 and SD of 1. (B) Venn diagram and (C) table showing the number of genes up- or down-regulated ≥twofold in rapamycin or proline medium conditions compared to YPD for the WT and *vps45* mutant. (D) Principal Component Analysis (PCA) showing distinct clustering for the *vps45* mutant and WT transcriptional programs upon a shift from YPD to proline-containing medium. (E) X-Y scatter plots showing genome-wide averaged transcript ratios following the shift from YPD to proline medium or rapamycin treatment between the WT and *vps45* mutant. Transcript ratios are colored green to represent greater than twofold increased induction or decreased repression, red to represent greater than twofold increased repression or decreased induction, and black to represent less than twofold difference in expression ratios, for the *vps45* mutant relative to the WT. Genes identified by this analysis are listed in Table 1, Table 2, and Table 3, as indicated.

We compared the gene expression profiles obtained using Affymetrix gene chip microarrays for the WT and *vps45* mutant grown in a rich nitrogen source medium (YPD), and following TORC1 inactivation by either 30 min rapamycin exposure or a shift from YPD into proline medium for 1 hr. Whereas the transcriptional profile of the WT and *vps45* strains were remarkably similar in the control untreated growth condition, we found substantial reprogramming of this profile under the TORC1-inhibiting growth conditions in the transcript levels of both strains (Figure 2A). Of the genes differentially expressed upon rapamycin treatment, 68% were induced and 88% were repressed >twofold in both the WT and *vps45* mutant. Of the genes differentially expressed upon a proline shift, 57% were induced and 49% were repressed >twofold by both strains (Figure 2, B and C). There was also a considerable overlap between genes differentially expressed upon both rapamycin treatment and a shift to proline medium (38.5% and 45.3% of genes for the WT and *vps45* mutant, respectively Figure 2, A–C). Interestingly, hierarchical clustering and principal component analysis of the data showed that while the WT and *vps45* transcript profiles cluster together in YPD and in the rapamycin treatment conditions, these profiles cluster separately from each other in proline medium (Figure 2, A and D). Significantly, by comparing the magnitude of transcript change from YPD shift to proline or rapamycin by scatter plot analysis, we noted only 18 genes for which the average rapamycin transcript ratios differed >twofold between the WT and *vps45* mutant. In contrast, this number increased to 63 genes when these strains were shifted from YPD to proline medium, including 12 of the genes that were also differentially expressed upon rapamycin exposure (Figure 2E, Table 1, Table 2, and Table 3). Taken together, our data indicate that perturbation of Golgi-to-vacuole trafficking has a greater effect on TORC1-regulated transcriptional programs following a shift from YPD to the poor nitrogen source proline than upon rapamycin treatment.

**Table 1:** Genes induced >twofold (fold-change in bold text) in the *vps45* mutant compared with the wild-type (WT) following a shift from YPD to proline (pro) medium

Gene	WT rapa/YPD	*vps45* rapa/YPD	Fold-Change (rapa)	WT pro/YPD	*vps45* pro/YPD	Fold-Change (pro)
Methionine/sulfur amino acid metabolism						
* MHT1*	0.31	0.51	1.67	3.04	9.25	**3.05**
* MET2*	0.82	1.37	1.67	6.92	21.03	**3.04**
* MMP1*	0.23	0.29	1.29	5.21	14.57	**2.80**
* STR3*	1.29	2.25	1.75	12.52	31.68	**2.53**
* DUG3*	0.59	0.80	1.34	1.33	3.19	**2.40**
* MET28*	3.83	4.84	1.26	8.55	19.21	**2.25**
* SEO1*	1.57	1.70	1.08	18.14	37.90	**2.09**
* MET32*	0.61	0.79	1.29	9.29	19.10	**2.06**
Mating/pheromone response						
* MFA2*	1.06	1.24	1.16	1.34	10.14	**7.56**
* AGA2*	1.25	1.04	1.20	1.36	8.89	**6.55**
* STE2*	2.01	1.74	1.16	2.53	8.66	**3.42**
* MFA1*	0.85	0.82	1.04	0.98	3.06	**3.13**
* BAR1*	1.47	1.27	1.16	1.18	3.00	**2.54**
Other						
* YHR210C*	2.01	2.72	1.35	1.09	2.34	**2.14**
* ARG1*	9.27	23.45	2.53	15.96	33.38	**2.09**

The expression following rapamycin (rapa) treatment relative to YPD levels is also shown.

**Table 2:** Genes expressed >twofold (fold-change in bold text) in the wild-type compared with the *vps45* mutant following a shift from YPD to proline (pro) medium

Gene	WT rapa/YPD	*vps45* rapa/YPD	Fold-Change (rapa)	WT pro/YPD	*vps45* pro/YPD	Fold-Change (pro)
NCR-controlled						
* PUT1*	10.80	3.90	2.77	23.74	4.84	**4.91**
* DUR3*	24.84	9.16	2.71	28.79	5.91	**4.87**
* OPT2*	9.36	10.66	1.14	9.26	2.49	**3.73**
* DAL80*	112.17	54.15	2.07	79.38	21.43	**3.70**
* PUT4*	26.71	12.45	2.14	16.02	4.77	**3.36**
* DAL5*	22.97	12.53	1.83	6.34	2.01	**3.16**
* MEP2*	27.29	12.31	2.22	19.12	7.10	**2.69**
* DAL4*	14.61	9.96	1.47	4.98	2.02	**2.47**
* GAP1*	24.93	11.81	2.11	23.96	10.39	**2.31**
* HAP4*	0.49	0.73	1.49	0.87	0.40	**2.19**
* GDH2*	2.84	2.71	1.05	4.01	1.91	**2.09**
* DAL7*	15.81	10.97	1.44	6.26	3.03	**2.07**
* DAL1*	11.27	10.39	1.09	4.50	2.21	**2.04**
* DUR1,2*	9.56	6.35	1.50	7.22	3.59	**2.01**
Phosphate-responsive						
* PHO89*	0.47	0.18	2.53	2.72	0.25	**10.76**
* SPL2*	0.48	0.34	1.41	1.24	0.25	**4.99**
* PHO5*	0.44	0.45	1.02	0.97	0.28	**3.48**
* PHM6*	0.24	0.29	1.21	0.72	0.28	**2.56**
* INM1*	0.47	0.48	1.02	2.02	0.96	**2.11**
* VTC3*	0.42	0.36	1.17	0.82	0.40	**2.03**
* PHO84*	0.57	0.19	2.95	1.19	0.59	**2.02**
* TMA10*	1.89	1.47	1.28	3.78	1.89	**2.00**
Ribosomal protein/ribosome biogenesis						
* DBP2*	0.13	0.17	1.31	0.30	0.14	**2.14**
* ERB1*	0.05	0.04	1.03	0.37	0.18	**2.10**
* RPA135*	0.06	0.09	1.50	0.47	0.23	**2.02**
* RPA190*	0.08	0.10	1.25	0.39	0.19	**2.01**
Hexose transporters						
* HXT2*	0.32	0.37	1.16	0.83	0.35	**2.38**
* HXT5*	5.63	2.14	2.63	12.99	6.47	**2.01**
* HXT6/HXT7*	0.87	0.55	1.58	0.70	0.35	**2.01**
Mating/pheromone response						
* MF(ALPHA)2*	0.87	0.56	1.56	0.83	0.14	**5.84**
* PRM7*	0.76	0.75	1.01	1.84	0.73	**2.53**
* SAG1*	0.99	0.77	1.28	0.94	0.37	**2.50**
Other						
* TPO3*	1.12	0.67	1.66	2.60	0.84	**3.08**
* OLE1*	1.26	1.18	1.07	1.50	0.51	**2.94**
* BAG7*	3.31	1.68	1.97	6.70	2.43	**2.75**
* YLR413W*	0.19	0.14	1.36	0.22	0.08	**2.75**
* NCA3*	1.16	0.57	2.04	4.10	1.50	**2.73**
* ECM13*	2.50	2.79	1.12	27.60	10.30	**2.68**
* MCH5*	1.13	1.54	1.36	9.33	3.52	**2.65**
* ISF1*	0.91	0.88	1.03	1.43	0.55	**2.58**
* YOR338W*	4.80	2.84	1.69	13.76	5.38	**2.56**
* GPG1*	9.01	3.44	2.62	9.56	3.92	**2.44**
* YPL088W*	6.60	3.11	2.12	6.12	2.52	**2.43**
* CTR1*	1.21	1.00	1.20	0.28	0.12	**2.39**
* INO1*	1.28	1.06	1.21	6.06	2.71	**2.23**
* GSC2*	1.87	1.14	1.63	3.26	1.47	**2.21**
* ARO9*	2.59	2.18	1.19	0.16	0.07	**2.17**
* FMP48*	1.25	1.02	1.22	3.50	1.65	**2.12**

The expression following rapamycin (rapa) treatment relative to YPD levels is also shown.

**Table 3:** Genes differentially expressed >twofold (fold-change in bolded text) between the wild-type and *vps45* mutant following 30 min rapamycin (rapa) treatment in YPD

Gene	WT rapa/YPD	*vps45* rapa/YPD	**Fold-Change (rapa)**	WT pro/YPD	*vps45* pro/YPD	Fold-Change (pro)
NCR-controlled						
* PUT1*	10.80	3.90	**2.77**	23.74	4.84	4.91
* DUR3*	24.84	9.16	**2.71**	28.79	5.91	4.87
* MEP2*	27.29	12.31	**2.22**	19.12	7.10	2.69
* PUT4*	26.71	12.45	**2.14**	16.02	4.77	3.36
* GAP1*	24.93	11.81	**2.11**	23.96	10.39	2.31
* DAL80*	112.17	54.15	**2.07**	79.38	21.43	3.70
Phosphate-responsive						
* PHO84*	0.57	0.19	**2.95**	1.19	0.59	2.02
* PHO89*	0.47	0.18	**2.53**	2.72	0.25	10.76
* CIT2*	4.24	1.88	**2.26**	0.79	0.83	1.05
Other						
* HXT5*	5.63	2.14	**2.63**	12.99	6.47	2.01
* GPG1*	9.01	3.44	**2.62**	9.56	3.92	2.44
* NQM1*	6.24	2.59	**2.40**	2.83	2.66	1.07
* SIP18*	5.13	2.25	**2.28**	1.97	1.78	1.11
* PIR3*	11.08	5.37	**2.06**	13.11	7.29	1.80
* NCA3*	1.16	0.57	**2.04**	4.10	1.50	2.73
* SNO1*	7.86	3.87	**2.03**	6.13	4.13	1.48
* YMR265C**^a^*	1.28	2.83	**2.21**	1.16	1.94	1.67
* ARG1**^a^*	9.27	23.45	**2.53**	15.96	33.38	2.09

The expression following proline (pro) treatment relative to YPD levels is also shown.

a Higher in *vps45* than WT following rapamycin treatment.

Gene sets for which the magnitude of YPD:rapamycin or YPD:proline transcript ratios differed >twofold between the WT and *vps45* mutant were enriched for six functional categories. These include methionine/sulfur amino acid metabolism, phosphate-responsive, and ribosomal protein/ribosome biogenesis (RP/Ribi) (addressed in individual sections below), as well as hexose transporter, mating/pheromone response, and NCR-controlled genes (Table 1, Table 2, and Table 3). To validate our earlier results (Puria *et al.* 2008), we compared the expression of genes classified as NCR genes. Of the 47 NCR responsive genes induced >twofold by rapamycin treatment in the WT, 13% show >twofold reduced induction in the *vps45* mutant (listed in Table 3). Moreover, 34% of the 41 NCR genes expressed >twofold by the WT upon shift into proline medium showed >twofold reduced induction in the *vps45* mutant (listed in Table 2). The finding that disruption of Golgi-to-endosome vesicular trafficking more extensively dampens NCR gene expression in response to a shift to proline medium compared with that seen in response to rapamycin inhibition corroborates our earlier reports (Puria *et al.* 2008).

Compared with the WT, mutation of *VPS45* also resulted in increased repression or reduced induction of certain hexose transporters in proline medium but not upon rapamycin treatment (Table 2). Hexose transporters are controlled by Rgt1 (Ozcan and Johnston 1999), and transcription of *HXT5* and other Rgt1 target genes is also controlled by the TORC1-regulated stress response element transactivators Msn2 and Msn4 (Beck and Hall 1999; Verwaal *et al.* 2002). However, we found that, except for *HXT5*, expression of the majority of the 180 genes reported to be activated by the stress response and/or dependent on Msn2/4, to be induced comparably between the WT and *vps45* mutant upon proline treatment [(Gasch *et al.* 2000; Causton *et al.* 2001) Table S7]. Similarly, we also found no evidence that, upon a shift into proline medium, *vps45* mutation alters expression of TORC1-regulated retrograde genes controlled by the Rtg1 and Rtg3 transcriptional activators (Dilova *et al.* 2002). Furthermore, while mating and pheromone-responsive genes were also highly differentially expressed upon a shift to proline medium in the *vps45* mutant compared with the WT strain (Table 1 and Table 2), the expression profile of these genes was not altered by rapamycin treatment in the *vps45* strain, or in the WT. Therefore, the mating pheromone response does not comprise a TORC1-regulated program that depends on Golgi-to-endosome trafficking status, but instead these results may be due to the stabilizing effects on pheromones and pheromone receptors caused by vesicular trafficking defects (Amerik *et al.* 2000).

### Golgi-to-endosome trafficking perturbation up-regulates methionine/sulfur amino acid metabolism genes in proline medium

**Figure 3:**
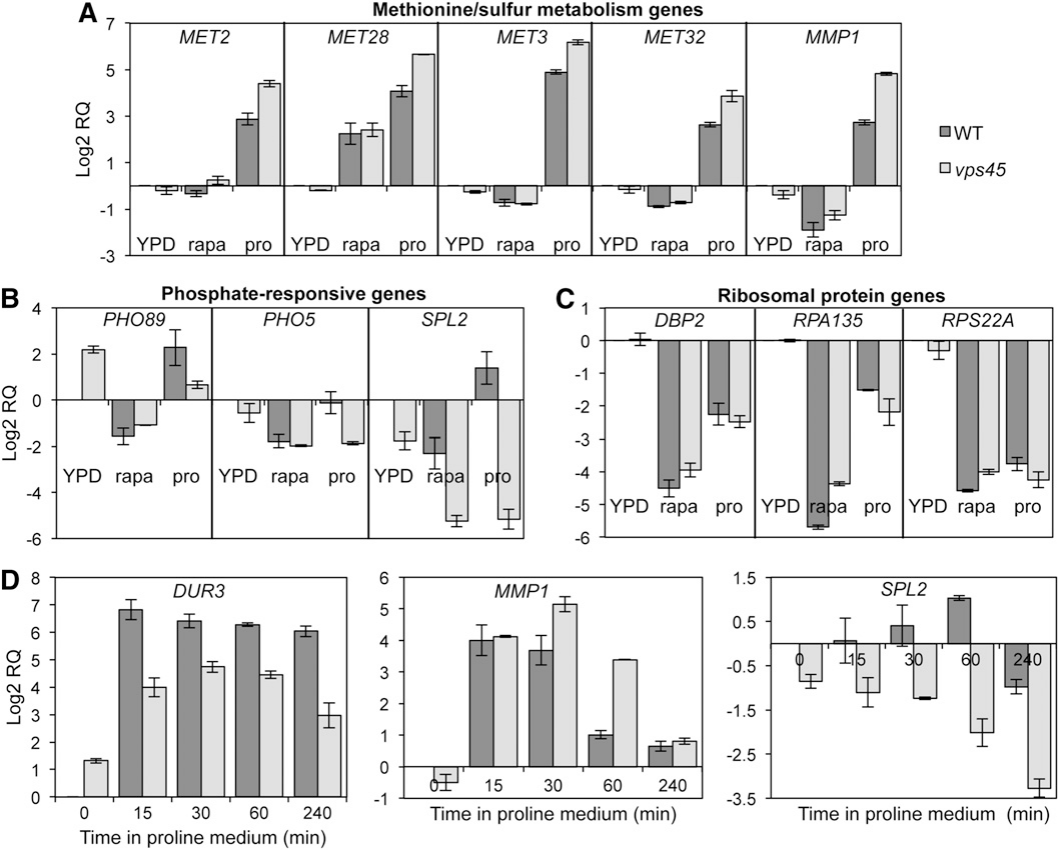
Mutation of *vps45* increases expression of methionine/sulfur amino acid metabolism genes, and decreases phosphate-responsive genes in proline-grown cells. Relative transcript levels of (A) *MET2*, *MET28*, *MET3*, *MET32*, and *MMP1*; (B) *PHO89*, *PHO5*, and *SPL2*; and (C) *DBP2*, *RPA135*, and *RPS22A* from YPD, YPD + 200 nM rapamycin, and S-proline-grown cultures of the WT and *vps45* strains were determined using quantitative RT-PCR. (D) Time-dependent expression of *DUR3*, *SPL2*, and *MMP1* expression following a shift from YPD to S-proline medium. For each experiment, transcript levels were normalized to *ACT1* levels and arbitrarily represented as a ratio to the gene-of-interest from the YPD-grown WT. Error bars depict the SDs from the mean of two independent experiments.

We find that expression of methionine and sulfur amino acid metabolism genes, controlled by the transcriptional activator Met4 [reviewed by (Ouni *et al.* 2011)], is significantly influenced by the *vps45* mutation. Although Met4-regulated genes were not regulated in a concerted manner by rapamycin, with 16% induced and 11% repressed >twofold, the majority of these genes were induced in S-proline medium in both the WT and the *vps45* mutant (Table S4). Interestingly, expression in response to a shift into proline medium was increased a further >twofold for eight methionine and sulfur amino acid metabolism genes in the *vps45* mutant (Table 1, Table S4, and Figure 3).

To further verify results obtained by microarray analysis, we examined the expression of a subset of the differentially regulated methionine/sulfur metabolism genes *MET2*, *MET28*, *MET3*, *MET32*, and *MMP1* genes using RT-PCR and found expression to be in agreement with the microarray results (Figure 3A). We also employed RT-PCR to follow expression kinetics of a representative methionine/sulfur metabolism gene *MMP1*, as well as the NCR gene *DUR3* as a comparison, following a shift from YPD to proline medium for 0, 15, 30, 60, and 240 min. First, the high-level expression of the NCR gene *DUR3* in response to the proline shift remained sustained throughout the experiment, and was consistently higher in the WT than in the *vps45* mutant at all time-points (Figure 3D). The *MMP1* gene was expressed at equally high levels between the WT and *vps45* mutant at early time points (15 min) following the proline shift (16.1- and 17.5-fold higher than WT expression in YPD, respectively) (Figure 3D). However, whereas WT *MMP1* expression had reduced to low levels after 60 min (2.0-fold higher expression than YPD), transcript levels in the *vps45* mutant remained highly expressed at this time (10.5-fold higher expression than WT YPD), although expression was reduced to WT levels after 240 min. These results suggest that, while the proline shift induces an early burst of methionine/sulfur metabolism gene induction, cells adjust expression to basal levels once cells have adapted to the growth conditions, and Golgi-to-endosome trafficking plays a role in this expression adjustment.

**Figure 4:**
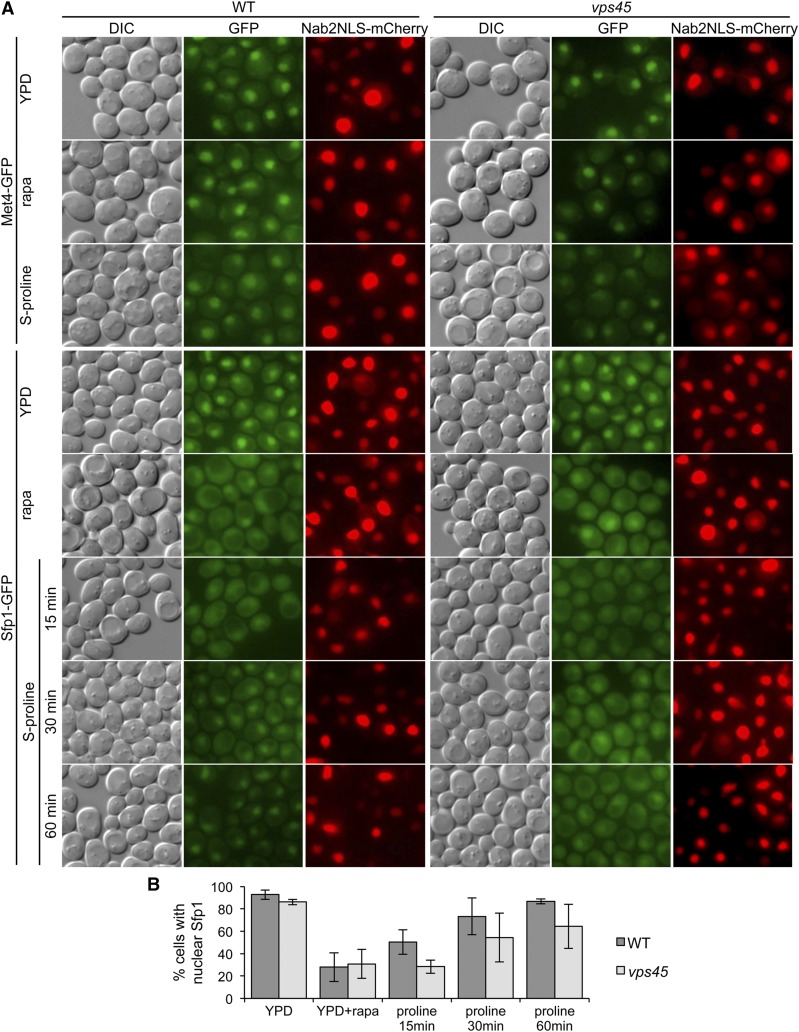
Effects of *vps45* mutation on nuclear localization of transcription factors. (A) Subcellular localization of Met4-GFP and Sfp1-GFP was visualized microscopically following growth of cells to log-phase in YPD medium, 30 min treatment with 100 nM rapamycin, or transfer to S-proline+his+ura+met for the times indicated. To determine nuclear localization, cells also expressed the nuclear marker Nab2-NLS-mCherry. GFP images for each transcription factor were photographed with identical exposure times and processed identically with regard to reduction of background signal and brightness contrast using ImageJ64 software. (B) The percentage of cells in which Sfp1-GFP colocalized with the Nab2-NLS-mCherry signal was quantified as described in *Materials and Methods*. Data were obtained from at least three fields of view, and averaged over at least two experiments.

We also examined if, as observed for Gln3, defects in Golgi-to-endosome trafficking may differentially affect the nuclear/cytoplasmic translocation of the methionine/sulfur metabolism gene transcription factor Met4 upon rapamycin treatment compared with a shift to proline medium. To test this hypothesis, we assessed the cellular localization of GFP-tagged Met4 in WT and the *vps45* mutant grown in YPD, followed by a 30-min rapamycin treatment (100 nM), or shifted into S-proline for 1 hr. In accordance with gene expression data (Table 1), we predicted increased nuclear localization for Met4 in proline relative to YPD, and in the *vps45* mutants relative to WT. However, we observed Met4-GFP to be predominantly nuclear in both strains, under all conditions tested (Figure 4A), contrasting with a previous report that rapamycin treatment induces Met4 eviction from the nucleus in a genome-wide protein localization study (Shin *et al.* 2009). Thus, the role of Golgi-to-endosome trafficking in methionine/sulfur metabolism gene expression adjustment following a proline shift is likely not mediated via Met4 localization. Taken together, our results are consistent with endolysosomal trafficking controlling NCR and methionine/sulfur metabolism gene transcriptional programs by distinct mechanisms.

Transcriptional activation by Met4 also depends on interactions with the DNA-binding factors Cbf1 or Met31 and Met32, and an additional cofactor, Met28, which stabilizes Met4-Cbf1 DNA-bound complexes (Kuras *et al.* 1996, 1997; Blaiseau *et al.* 1997; Lee *et al.* 2010). Interestingly, we find that the positive effector genes *MET28* and *MET32* are themselves up-regulated in the *vps45* mutant relative to the WT in proline medium (Table 1 and Figure 3A), which could contribute to an elevated methionine/sulfur metabolism transcriptional response, although as *MET28* is itself regulated by this pathway (Lee *et al.* 2010), we cannot differentiate between cause and effect. High intracellular levels of cysteine act as the sensor to negatively regulate Met4 by the SCF-Met30 ubiquitin ligase, through ubiquitination of Met4 by SCF-Met30 (reviewed by Chandrasekaran and Skowyra 2008; Ouni *et al.* 2011). Because Vps-D mutants exhibit substantially lower expression levels of the NCR-regulated general amino acid permease Gap1 (Puria *et al.* 2008), it is likely that cysteine levels are reduced in the Vps-D mutant, resulting in reduced Met4 ubiquitination and inhibition. Alternatively, perturbation of Golgi-to-endosome trafficking may instead affect Met4 ubiquitination, phosphorylation, and/or turnover, thereby increasing promoter occupancy of target genes. Intriguingly, hyperactivation of the Met4 transcriptional response has also been reported to repress hexose transporter genes (Lee *et al.* 2010), and this may account for the observed downregulation of these genes upon perturbation of Golgi-to-endosome trafficking.

### Phosphate-responsive genes are downregulated in proline medium by Golgi-to-endosome trafficking disruption

Phosphate-responsive genes controlled by the transcriptional activator Pho4 (O’Neill *et al.* 1996; Springer *et al.* 2003) comprise another functional class that we found to be differentially regulated in the *vps45* mutant. The majority of the phosphate-responsive genes identified were repressed upon rapamycin treatment in both strains, and either induced or repressed in the WT in proline, while the *vps45* mutation caused >twofold repression of these genes (or attenuated induction for *TMA10*) relative to the WT in proline (Table 2). We also found three genes in this category that were more repressed upon rapamycin treatment in the *vps45* mutant (Table 3). The expression patterns of the three phosphate-responsive genes (*PHO89*, *PHO5*, and *SPL2*) that were most differentially expressed upon a shift to proline medium were further verified by RT-PCR, and were in agreement with microarray results (Figure 3B). The *SPL2* expression kinetics were also monitored following a shift from YPD to proline medium. While we observe a twofold increase in WT *SPL2* expression over 1 hr, the *vps45* mutant *SPL2* levels consistently declined over the same time period, and were reduced to approximately one-tenth of the WT YPD reference levels after 4 hr (Figure 3D).

Downregulation of the phosphate-responsive genes upon rapamycin treatment is consistent with Pho4-regulated genes being subject to TORC1 control, and this is in accord with previous microarray studies reporting the response of WT yeast cells to rapamycin (Cardenas *et al.* 1999; Hardwick *et al.* 1999). Although we did not identify the precise mechanism via which disruption of Golgi-to-endosome trafficking inhibits phosphate-responsive gene transcription, our results add to an already appreciated and intricate association between phosphate signaling and vesicular machinery. The vacuole comprises the major storage site for inorganic polyphosphate (Urech *et al.* 1978; Saito *et al.* 2005), which is integral for buffering of intracellular phosphate levels (Thomas and O’Shea 2005). As such, vacuolar transporter chaperone complex genes are important for phosphate homeostasis, and are also targets of the phosphate-responsive regulon (Ogawa *et al.* 2000; Boer *et al.* 2003; Secco *et al.* 2012).

### Golgi-to-endosome trafficking disruption decreases RP/Ribi gene expression and delays Sfp1 nuclear relocalization following nitrogen downshift

TORC1 inhibition by rapamycin results in repression of the RP/Ribi genes regulated by the transcription factor Sfp1 (Powers and Walter 1999; Martin *et al.* 2004; Jorgensen *et al.* 2004; Lempiainen *et al.* 2009; Marion *et al.* 2004). Remarkably, of 149 genes annotated as ribosomal proteins (Table S5) and 235 genes categorized as Ribi genes [Table S6 and Jorgensen *et al.* (2004)], we identified only four that were differentially expressed >twofold between the WT and *vps45* mutant (Table 2). Interestingly however, the majority of RP/Ribi genes also showed some degree of greater repression in proline in the *vps45* mutant, with 34% of genes repressed ≥1.5 fold more than in the WT strain (Table S5 and Table S6). In contrast, only 0.5% of the same genes were ≥1.5 fold more repressed in the *vps45* mutant than the WT strain upon rapamycin treatment (Table S5 and Table S6). Furthermore, three candidate RP/Ribi genes (*DBP2*, *RPA135* and *RPS22A*) were modestly more repressed in the proline-grown *vps45* mutant relative to the WT when assayed by RT-PCR (Figure 3C). In growing cells, a majority of the total cellular transcriptional activity is directed toward ribosome biogenesis, which is a major determinant of cell growth rate (Warner 1999). This, together with the very short half-life of RP/Ribi transcripts, results in RP/Ribi transcript abundance directly mirroring transcription rate (Warner 1999; Grigull *et al.* 2004). Thus, even small changes in ribosome biogenesis likely have significant effects on the cell, such as those required to adapt to a nutritional downshift by economizing and diverting metabolism resources away from active protein synthesis.

We next examined the possible effect of the *vps45* mutation on Sfp1 nuclear localization. Based on RP/Ribi gene expression (Table 2, Table S5, and Table S6), and a demonstrated cytoplasmic relocalization upon rapamycin treatment (Marion *et al.* 2004), we predict the maximum nuclear localization of Sfp1 in YPD-grown cells, followed by proline-grown WT, proline-grown *vps45* mutant, and greatest cytoplasmic localization upon rapamycin treatment. Sfp1-GFP signal was predominantly nuclear in YPD-grown cells (Figure 4; nuclear foci in 93% of WT and 86% of *vps45* cells), and largely cytoplasmic following rapamycin treatment, which completely halts growth (Figure 4; nuclear foci in 28% of WT and 31% of *vps45* cells). Similarly, Sfp1-GFP translocated to the cytoplasm at early time-points following a shift to growth-limiting proline medium (15 min; 50% and 28% nuclear localization for the WT and *vps45* mutant, respectively, Figure 4), but relocated back to the nucleus as cells adapted to growth in proline medium. Interestingly, in contrast to the WT, in the *vps45* mutant there was a marked delay in nuclear Sfp1 relocalization following a shift to proline medium (Figure 4). Therefore, our results indicate that Golgi-to-endosome trafficking contributes to recovery of RP/Ribi gene expression by controlling expedited Sfp1 relocalization back to the nucleus following a downshift to the poor nitrogen source, proline.

In favorable growth conditions, TORC1 promotes RP/Ribi gene transcription by directly binding to and phosphorylating Sfp1, thereby enabling Sfp1 nuclear translocation (Lempiainen *et al.* 2009), which then drives transcription together with Ifh1/Fhl1 (Jorgensen *et al.* 2004; Rudra *et al.* 2005). Interestingly, rapamycin treatment but not nutrient depletion reduces Sfp1 phosphorylation and Sfp1-TORC1 interaction, even though both conditions are capable of driving Sfp1 out of the nucleus (Lempiainen *et al.* 2009). Sfp1 is retained in the cytoplasm during both rapamycin treatment and nitrogen limitation, and this requires the Rab escort protein Mrs6 in a poorly understood, TORC1-dependent mechanism (Singh and Tyers 2009; Lempiainen *et al.* 2009). Consistent with our results indicating an involvement of the Vps-D complex in the control of Sfp1 nuclear import, Mrs6 is also required to promote prenylation and membrane delivery of GTPases that promote Golgi-to-Golgi and ER-to-Golgi vesicular transport (Benito-Moreno *et al.* 1994; Jiang and Ferro-Novick 1994). Additionally, other components of the secretory and vesicular trafficking pathways also influence Sfp1 nuclear/cytoplasmic trafficking (Singh and Tyers 2009), and treatment with the secretory pathway inhibitor tunicamycin results in both reduced Sfp1 phosphorylation (Lempiainen *et al.* 2009) and cytoplasmic retention/reduced nuclear localization of Sfp1 (Jorgensen *et al.* 2004; Singh and Tyers 2009).

Taken together, our results show that Golgi-to-endosome trafficking plays distinct and varied roles in the regulation of multiple TORC1-regulated transcriptional programs in response to a nutritional downshift. Given the eukaryotic conservation of TORC1 and endomembrane networks, analogous mechanisms may also control TORC1-mediated responses to nutritional cues in mammals.

## Supplementary Material

Supporting Information
