# Supplementary material for: Vesicular Trafficking Systems Impact TORC1-Controlled Transcriptional Programs in Saccharomyces cerevisiae
Source: G3 (Bethesda). 2016 Jan 6;6(3):641–52. doi: 10.1534/g3.115.023911 (PMC4777127; doi:10.1534/g3.115.023911)
Supplement: Supporting Information [file supp_6_3_641__index.html]

Vesicular Trafficking Systems Impact TORC1-Controlled Transcriptional Programs in Saccharomyces cerevisiae — Supporting Information 

# Vesicular Trafficking Systems Impact TORC1-Controlled Transcriptional Programs in *Saccharomyces cerevisiae*

## Supporting Information for Kingsbury and Cardenas, 2016

**Files in this Data Supplement:**

- Table S1 - Strains used in this study. (.docx, 24 KB)
- Table S2 - Plasmids used in this study. (.docx, 14 KB)
- Table S3 - Primers used in this study. (.docx, 15 KB)
- Table S4 - Expression data of methionine/sulfur amino acid metabolism genes. (.docx, 22 KB)
- Table S5 - Microarray expression results for ribosomal protein genes. (.xlsx, 23 KB)
- Table S6 - Microarray expression results for ribosome biogenesis (RIBI) genes. (.xlsx, 35 KB)
- Table S7 - Microarray expression results for stress response genes. (.xlsx, 27 KB)
- Table S8 - ANOVA comparison of all expression data. (.xlsx, 4829 KB)
