# Supplementary material for: Vesicular Trafficking Systems Impact TORC1-Controlled Transcriptional Programs in Saccharomyces cerevisiae
Source: G3 (Bethesda). 2016 Jan 6;6(3):641–52. doi: 10.1534/g3.115.023911 (PMC4777127; doi:10.1534/g3.115.023911)
Supplement: Supporting Information [file supp_g3.115.023911_TableS1.docx]

**Table S1:** **Strains used in this study.** Strains were isogenic with BY4742 or BY4741, as indicated.

| **Strain** | **Genotype** | **Source/Reference** |
| --- | --- | --- |
| BY4741 | *MAT***a** *his3*∆*1* *leu2*∆*0* *met15*∆*0* *ura3*∆*0* | ([Brachmann et al. 1998](#_ENREF_1)) |
| BY4742 | *MAT*α *his3*Δ*1* *leu2*Δ*0 lys2*Δ*0* *ura3*Δ*0* | ([Brachmann et al. 1998](#_ENREF_1)) |
| YJK3814 | BY4742 [pPC10] [pJK28] | ([Kingsbury et al. 2014](#_ENREF_2)) |
| YJK3815 | BY4742 *pep3*∆::kanMX4 [pPC10] [pJK28] | ([Kingsbury et al. 2014](#_ENREF_2)) |
| Y4853 | BY4742 *vps45*∆::kanMX4 | Yeast Deletion Collection |
| YJK3855 | BY4741 *MET4*-GFP-*HIS3*MX [pNab2-NLS] | This study |
| YJK4060 | BY4742 *vps45*∆::kanMX4 [pPC10] [pJK28] | This study |
| YJK4095 | BY4742 *PHO4*-GFP-kanMX6 [pNab2-NLS] | This study |
| YJK4096 | BY4742 *PHO4*-GFP-kanMX6 *vps45*∆::natMX4 [pNab2-NLS] | This study |
| YJK4115 | BY4741 *MET4*-GFP-*HIS3*MX *vps45*∆::kanMX4 [pNab2-NLS] | This study |
| YJK4146 | BY4741 *SFP1*-GFP-*HIS3*MX *vps45*∆::kanMX4 [pNab2-NLS] | This study |
| YJK4147 | BY4741 *SFP1*-GFP-*HIS3*MX [pNab2-NLS] | This study |
