# Supplementary material for: Vesicular Trafficking Systems Impact TORC1-Controlled Transcriptional Programs in Saccharomyces cerevisiae
Source: G3 (Bethesda). 2016 Jan 6;6(3):641–52. doi: 10.1534/g3.115.023911 (PMC4777127; doi:10.1534/g3.115.023911)
Supplement: Supporting Information [file supp_g3.115.023911_TableS2.docx]

**Table S2: Plasmids used in this study.**

| Plasmid | Description | Source/Reference |
| --- | --- | --- |
| pNab2-NLS | pNab2-NLS (TPI1::Nab2-NLS-2mcherry)/*LEU2* | M. Rout |
| pPC10 | *CEN6* *HIS3 HA_3_-SCH9* | ([Kingsbury et al. 2014](#_ENREF_2)) |
| pJK28 | *CEN6* *URA3 LYS2 LEU2* | ([Kingsbury et al. 2014](#_ENREF_2)) |
