# Supplementary material for: Vesicular Trafficking Systems Impact TORC1-Controlled Transcriptional Programs in Saccharomyces cerevisiae
Source: G3 (Bethesda). 2016 Jan 6;6(3):641–52. doi: 10.1534/g3.115.023911 (PMC4777127; doi:10.1534/g3.115.023911)
Supplement: Supporting Information [file supp_g3.115.023911_TableS3.docx]

**Table S3. Primers used in this study.**

| **Name** | **Sequence (5’-3’)^*^** | **Purpose**^‡^ |
| --- | --- | --- |
| JK5 | GTATTCTGGGCCTCCATGTC | MX4 cassette, B |
| JK6 | GACATCATCTGCCCAGATGC | MX4 cassette, B |
| JK177 | TTTGGAACTGGGCATAAAGC | *MET4*, B |
| JK178 | AACCCGGGGATCCGTCGACC | GFP*, B* |
| JK179 | ACGGTGGTATTCTACAAGAC | *MET3*, C |
| JK187 | CAGAGGAGTAATCGTTTTCG | *MET3*, C |
| JK243 | AGCAAACCCCAAGATCGTAG | *MET32*, C |
| JK244 | GTCCTGTTTCTCCTACATGTCAG | *MET32*, C |
| JK247 | CGAACACTCCCTACAAATCCAC | *MET2*, C |
| JK248 | TCACTTCACCCGTTACAGAAC | *MET2*, C |
| JK255 | GTTCTCATTGCAGGTGGTGG | *MMP1*, C |
| JK256 | TCCAGAACGTACCTTTCGCT | *MMP1*, C |
| JK259 | GAATTGAGAGTTGCCCCAGA | *ACT1*, C |
| JK260 | AGAAGGCTGGAACGTTGAAA | *ACT1*, C |
| JK268 | CGCTTGCTATCATTGCTGCT | *MET28*, C |
| JK269 | TCTTTCTTCTCCGCTCTTGC | *MET28*, C |
| JK275 | TTTTGAGTTAAGGCCATCTTTTACTGTATAGAACAAAGAACAGCTGAAGCTTCGTACGC | *VPS45*, A |
| JK276 | TCATATATAAAATAGAATTTTAGAATAAGATAATCCTTATGCATAGGCCACTAGTGGATCTG | *VPS45*, A |
| JK284 | AAGGCAACACCACCATAAGC | *PHO89*, C |
| JK285 | AAGCCATTACTGCAGCCACT | *PHO89*, C |
| JK288 | AAACCACTTTTGCCAACTCG | *PHO5*, C |
| JK289 | CGGATTCAGCTTCACTGACA | *PHO5*, C |
| JK292 | ACGCTGCGCTCTACTTGAAT | *SPL2*, C |
| JK293 | GGCAAAATGGAAACAGCAG | *SPL2*, C |
| JK298 | CTGCCGGTACATCCGTCACCTACAGCAGAACGTGAGCACGGGTCGACGGATCCCCGGGTT | *PHO4*, A |
| JK299 | AGTCCGATATGCCCGGAACGTGCTTCCCATTGGTGCACGGTCGATGAATTCGAGCTCGTT | *PHO4*, A |
| JK302 | GGATATGGACTGGATGATGC | *PHO4*, B |
| JK306 | TCCTCGTAGTGGTGCACAAG | *PHO4*, B |
| JK308 | AACGAGCTCGAATTCATCGA | GFP, B |
| JK309 | TTTTGGGCCCTAGTAACCAC | *SFP1*, B |
| JK318 | CAACGTGCATGGTAATTCAC | *SFP1*, B |
| JK321 | ACAGACCACAAGGCGGTAAC | *DBP2*, C |
| JK322 | TCTCACTGTCCGATCTGTCG | *DBP2*, C |
| JK325 | TACCCCAGCCGATTACTTTG | *RPA135*, C |
| JK326 | ACAGGACCAGTGGAACGAAC | *RPA135*, C |
| JK329 | TCAGACCATCCTCCAAGGTC | *RPS22A*, C |
| JK330 | CCAGCAGAGGTGGTCAAGAT | *RPS22A*, C |
| NDMC79 | ACGAGTACTGACTGTATG | *VPS45*, A |
| NDMC80 | ATATAGTGTAACGGCTATCAC | *VPS45*, A |
| NDMC81 | TATCACATGCGGTGTAAG | *VPS45*, B |

^*^ Underlined sequence is homologous to vector or cassette sequence

^‡^A. Disruption/insertion/mutation of designated gene; B. Verification of designated gene mutation/construct; C. RT-PCR.
