# Supplementary material for: Vesicular Trafficking Systems Impact TORC1-Controlled Transcriptional Programs in Saccharomyces cerevisiae
Source: G3 (Bethesda). 2016 Jan 6;6(3):641–52. doi: 10.1534/g3.115.023911 (PMC4777127; doi:10.1534/g3.115.023911)
Supplement: Supporting Information [file supp_g3.115.023911_TableS4.docx]

**Table S4:** **Expression data of methionine/sulfur amino acid metabolism genes.** Of the 44 Met4 core regulon genes, expression in S-proline (pro)/YPD media is on average 1.54 (0.48 SD) times higher in the *vps45* mutant compared with the WT, and ratios ≥2 are bolded. Expression in rapamycin (rapa)/YPD media is 1.10 (0.22 SD) times higher in the *vps45* mutant compared with the WT.

| *Gene* | *WT rapa/YPD* | *vps45 rapa/YPD* | *vps45/WT*  *rapa ratio* | *WT pro/YPD* | *vps45 pro/YPD* | *vps45/WT pro ratio* | *key MET regulator* | *Met4-regulated^ab^* | *Cbf1-binding site^a^* | *Met31/Met32 binding site^a^* |
| --- | --- | --- | --- | --- | --- | --- | --- | --- | --- | --- |
| *MET2* | 0.82 | 1.37 | 1.67 | 6.92 | 21.03 | **3.03** |  | 2 | + | + |
| *MMP1* | 0.23 | 0.29 | 1.28 | 5.21 | 14.57 | **2.78** |  | 3 | - | + |
| *STR3* | 1.29 | 2.25 | 1.75 | 12.52 | 31.68 | **2.50** |  | 3 | - | + |
| *DUG3* | 0.59 | 0.80 | 1.33 | 1.33 | 3.19 | **2.38** |  | 2 | - | + |
| *MET28* | 3.83 | 4.84 | 1.27 | 8.55 | 19.21 | **2.27** | + | 2 | + | + |
| *SEO1* | 1.57 | 1.70 | 1.09 | 18.14 | 37.90 | **2.08** |  | 3 | - | + |
| *MET32* | 0.61 | 0.79 | 1.30 | 9.29 | 19.10 | **2.04** | + |  |  |  |
| *MXR1* | 0.68 | 0.63 | 0.93 | 2.89 | 5.78 | **2.00** |  | 3 | - | + |
| *MET8* | 0.58 | 0.70 | 1.20 | 4.70 | 9.30 | 1.96 |  | 1 | + | - |
| *BDS1* | 1.26 | 1.51 | 1.20 | 2.02 | 3.85 | 1.92 |  | 2 | - | + |
| *MET1* | 1.25 | 1.51 | 1.20 | 8.59 | 15.54 | 1.82 |  | 1 | + | - |
| *RAD59* | 1.15 | 1.23 | 1.06 | 5.98 | 10.84 | 1.82 |  | 1 | + | + |
| *MUP1* | 0.09 | 0.13 | 1.45 | 1.47 | 2.63 | 1.79 |  | 3 | - | + |
| *YOL162W* | 2.57 | 1.59 | 0.62 | 8.94 | 14.71 | 1.64 |  | 3 | - | + |
| *MET30* | 1.40 | 1.86 | 1.33 | 2.46 | 3.77 | 1.54 | + | 1 | + | + |
| *SAM3* | 1.00 | 1.03 | 1.03 | 2.45 | 3.73 | 1.52 |  | 3 | - | + |
| *SER33* | 0.87 | 0.85 | 0.98 | 2.61 | 3.94 | 1.52 |  | 2 | + | + |
| *ICY2* | 3.72 | 4.64 | 0.80 | 5.63 | 8.44 | 1.50 |  | 1 | + | + |
| *MET5* | 0.82 | 0.92 | 1.12 | 4.86 | 7.28 | 1.49 |  | 2 | + | - |
| *MET22* | 1.56 | 1.74 | 1.11 | 2.91 | 4.20 | 1.45 |  | 1 | + | - |
| *ZWF1* | 1.42 | 1.50 | 1.06 | 2.17 | 3.15 | 1.45 |  | 2 | - | + |
| *MET3* | 1.11 | 1.42 | 1.28 | 14.28 | 20.63 | 1.45 |  | 3 | + | + |
| *MET17* | 0.79 | 0.84 | 1.06 | 2.38 | 3.37 | 1.41 |  | 3 | + | + |
| *GRX8* | 0.69 | 0.65 | 0.94 | 5.86 | 8.21 | 1.41 |  | 2 | - | + |
| *GSH1* | 1.02 | 1.01 | 0.98 | 2.17 | 2.98 | 1.37 |  | 2 | + | + |
| *FMO1* | 1.82 | 1.97 | 1.08 | 4.53 | 6.14 | 1.35 |  | 1 | - | - |
| *BNA3* | 1.48 | 1.59 | 1.08 | 2.43 | 3.22 | 1.33 |  | 2 | + | + |
| *SUL2* | 1.43 | 1.63 | 1.14 | 13.31 | 17.63 | 1.33 |  | 2 | + | + |
| *SAM1* | 0.15 | 0.20 | 1.41 | 1.55 | 2.02 | 1.30 |  | 3 | - | + |
| *SAM2* | 0.60 | 0.62 | 1.04 | 2.82 | 3.64 | 1.28 |  | 3 | + | + |
| *MET10* | 1.13 | 1.48 | 1.30 | 9.34 | 11.89 | 1.27 |  | 2 | + | + |
| *MET31* | 0.68 | 0.80 | 1.18 | 0.64 | 0.80 | 1.25 | + |  |  |  |
| *MET6* | 0.75 | 0.78 | 1.04 | 2.01 | 2.49 | 1.23 |  | 3 | + | + |
| *NIT1* | 4.42 | 4.33 | 0.98 | 7.25 | 8.86 | 1.22 |  | 2 | - | + |
| *YLL058W* | 1.82 | 1.65 | 0.91 | 1.82 | 2.23 | 1.22 |  | 1 | - | + |
| *CYS3* | 0.69 | 0.71 | 1.03 | 1.61 | 1.98 | 1.22 |  | 3 | + | + |
| *YHR112C* | 2.20 | 1.94 | 0.88 | 3.33 | 4.00 | 1.20 |  | 1 | + | + |
| *MET16* | 2.62 | 2.55 | 0.97 | 11.19 | 13.10 | 1.18 |  | 1 | + | - |
| *OPT1* | 0.48 | 0.49 | 1.02 | 1.42 | 1.66 | 1.16 |  | 3 | + | + |
| *CYS4* | 0.76 | 0.71 | 0.93 | 1.22 | 1.34 | 1.10 |  | 2 | + | + |
| *SUL1* | 4.70 | 4.30 | 0.92 | 125.58 | 135.97 | 1.09 |  | 2 | - | - |
| *ADE3* | 1.37 | 1.31 | 0.96 | 1.13 | 1.23 | 1.09 |  | 1 | + | + |
| *CBF1* | 1.01 | 1.13 | 1.11 | 1.03 | 1.11 | 1.09 | + |  |  |  |
| *ADI1* | 1.38 | 1.13 | 0.81 | 2.68 | 2.79 | 1.04 |  | 2 | - | + |
| *MET14* | 0.57 | 0.67 | 1.16 | 6.15 | 6.13 | 1.00 |  | 1 | - | + |
| *YCT1* | 1.96 | 1.77 | 0.90 | 7.76 | 7.63 | 0.98 |  | 2 | + | + |
| *HIT1* | 0.47 | 0.44 | 0.93 | 0.75 | 0.73 | 0.98 |  | 2 | - | + |
| *MET4* | 1.42 | 1.48 | 1.04 | 1.57 | 1.51 | 0.96 | + |  |  |  |

^a^ Data from ([Lee et al. 2010](#_ENREF_3)).

^b^ Transcription dependencies of Met4 core regulon for Cbf1 and Met28: Class 1, transcription strictly dependent on Met4 hyperactivation and in sulfur starvation; Class 2, intermediate dependence, important in sulfur starvation but not Met4 hyperactivation; Class 3, transcription independent of Cbf1 and Met28 ([Lee et al. 2010](#_ENREF_3)).

^c^ Data from ([Natarajan et al. 2001](#_ENREF_4))

^d^ ID, Insufficient Data

**REFERENCES**

Brachmann, C.B., A. Davies, G.J. Cost, E. Caputo, J. Li *et al.*, 1998 Designer deletion strains derived from *Saccharomyces cerevisiae* S288C: a useful set of strains and plasmids for PCR-mediated gene disruption and other applications. *Yeast* 14:115-132.

Kingsbury, J.M., N.D. Sen, T. Maeda, J. Heitman, and M.E. Cardenas, 2014 Endolysosomal membrane trafficking complexes drive nutrient-dependent TORC1 signaling to control cell growth in *Saccharomyces cerevisiae*. *Genetics* 196:1077-1089.

Lee, T.A., P. Jorgensen, A.L. Bognar, C. Peyraud, D. Thomas *et al.*, 2010 Dissection of combinatorial control by the Met4 transcriptional complex. *Molecular Biology of the Cell* 21:456-469.

Natarajan, K., M.R. Meyer, B.M. Jackson, D. Slade, C. Roberts *et al.*, 2001 Transcriptional profiling shows that Gcn4p is a master regulator of gene expression during amino acid starvation in yeast. *Mol Cell Biol* 21:4347-4368.
